# “Nonparametric Local Smoothing” is not image registration

**DOI:** 10.1186/1756-0500-5-610

**Published:** 2012-11-01

**Authors:** Torsten Rohlfing, Brian Avants

**Affiliations:** 1 Neuroscience Program, SRI International, 333 Ravenswood Avenue, CA 94025, Menlo Park, USA; 2 Penn Image Computing and Science Laboratory (PICSL), Department of Radiology, University of Pennsylvania School of Medicine, PA 19104, Philadelphia, USA

**Keywords:** Image registration, Correspondence, Accuracy

## Abstract

**Background:**

Image registration is one of the most important and universally useful computational tasks in biomedical image analysis. A recent article by Xing & Qiu (IEEE Transactions on Pattern Analysis and Machine Intelligence, 33(10):2081–2092, 2011) is based on an inappropriately narrow conceptualization of the image registration problem as the task of making two images look alike, which disregards whether the established spatial correspondence is plausible. The authors propose a new algorithm, Nonparametric Local Smoothing (NLS) for image registration, but use image similarities alone as a measure of registration performance, although these measures do not relate reliably to the realism of the correspondence map.

**Results:**

Using data obtained from its authors, we show experimentally that the method proposed by Xing & Qiu is not an effective registration algorithm. While it optimizes image similarity, it does not compute accurate, interpretable transformations. Even judged by image similarity alone, the proposed method is consistently outperformed by a simple pixel permutation algorithm, which is known by design not to compute valid registrations.

**Conclusions:**

This study has demonstrated that the NLS algorithm proposed recently for image registration, and published in one of the most respected journals in computer science, is not, in fact, an effective registration method at all. Our results also emphasize the general need to apply registration evaluation criteria that are sensitive to whether correspondences are accurate and mappings between images are physically interpretable. These goals cannot be achieved by simply reporting image similarities.

## Discussion

Image registration is one of the most commonly encountered and important problems in biomedical image analysis [1-7]. It is “a process for determining the correspondence of features between images” [6], “the determination of a one-to-one mapping or transformation between the coordinates in one space and those in another” [8], and with the objective to “bring the modalities involved into spatial alignment” [2]. Registration ”geometrically aligns two images” [9], thus “determining the spatial alignment between images” [4].

What is *not* the goal of registration, however, is to make one (“moving”) image appear maximally like another (“fixed” or “reference”) image. If this were the case, nothing would be gained by the process of registration, as we are given *a priori* the fixed image. Instead, the primary result of registration is the correspondence established between the images, and maximization of image similarity is a by-product of the accuracy of this correspondence. A useful geometric transformation between images must allow *interpretation* of their differences and, potentially, statistics in a well-defined transformation space. Performance measures of image registration, therefore, must consider the specific properties of correspondences in addition to the numerical similarity achieved between the images, especially when the transformation itself is the object of further analysis, as is the case for the widely used Tensor-Based or Deformation-Based Morphometry [10] methods.

### Nonparametric Local Smoothing (NLS)

In their recent paper, “Intensity-based image registration by Nonparametric Local Smoothing,” Xing & Qiu [11] state, incorrectly, that “the major goal of image registration is to find a geometrical transformation **T**(*x*,*y*)=(*T*_1_(*x*,*y*),*T*_2_(*x*,*y*)) such that *Z*_*M*_(**T**(*x*,*y*)) is as close to *Z*_*R*_(*x*,*y*) as possible.” (Herein, *Z*_*R*_ is a two-dimensional fixed image and *Z*_*M*_ the moving image being registered to *Z*_*R*_via transformation **T**(*x*,*y*).)

Solving this type of optimization problem is common in image registration, but it is not the goal in and of itself. Instead, the fundamental goal of image registration is to find a transformation **T** such that the difference ||**T**−**T**_true_||between estimated and true transformation is minimized. This, however, cannot be determined based on image similarity, even when, as Xing & Qiu constrain, the moving image is “a geometrically altered version of [the fixed image].”

This misconception of the purpose of image registration, however, is the foundation of their experiments that rely on image similarity alone to quantify registration quality. But image similarity is not a valid measure of registration accuracy [12], thus leaving unsupported the conclusion that “Nonparametric Local Smoothing” (NLS) performs effective image registration.

In this communication we provide *direct* and *specific* evidence that the experimental design and reasoning employed by Xing & Qiu [11] are flawed and that their NLS algorithm is not suitable for image registration at all by experimentally substantiating two crucial observations. Firstly, we show that a deformation field computed by the NLS method is largely arbitrary and substantially different from the ground truth. Second, to explain why the NLS algorithm appeared to achieve superior registrations, we show that all evaluation criteria used by Xing & Qiu are unsuitable to validate image registration, as a simple permutation-based algorithm outperforms the NLS method without achieving any meaningful alignment at all.

### The NLS method does not create interpretable transformations

**Figure 1:**
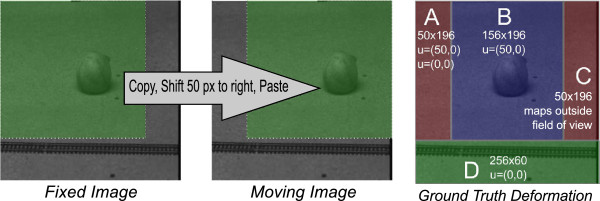
**Procedure to generate ground truth deformation field.** Illustration of reconstructed procedure to generate moving image (*left*) and corresponding ground truth deformation field (*right*) for “Ball” example. Based on careful inspection of the images provided to us, we determined that the top-left area of the fixed image had been copied and shifted to the right by 50 pixels. Thus, the ground truth deformation field comprises four distinct regions as shown on the right. The “moving” and “fixed” images were kindly provided by C. Xing and are reproduced with permission from IEEE (see Acknowledgements).

We obtained the identical images used in Ref. [11] and shown in Figures two, six, eight, and ten therein. From the images of the “Ball” example, we reconstructed the ground truth transformation between them as follows. We determined that the top-left area, 156×196 pixels, of the fixed image had been copied, shifted by 50 pixels, and pasted into the top-right image corner (Figure 1). The ground truth deformation field, *u*, is thus partitioned into four distinct regions: “A,” duplicated content, two equally correct mappings, *u*=(0,0)and *u*=(50,0); “B,” *u*=(50,0). “C,” content pasted over, transformation undefined; “D,” unmoved, *u*=(0,0).

**Figure 2:**
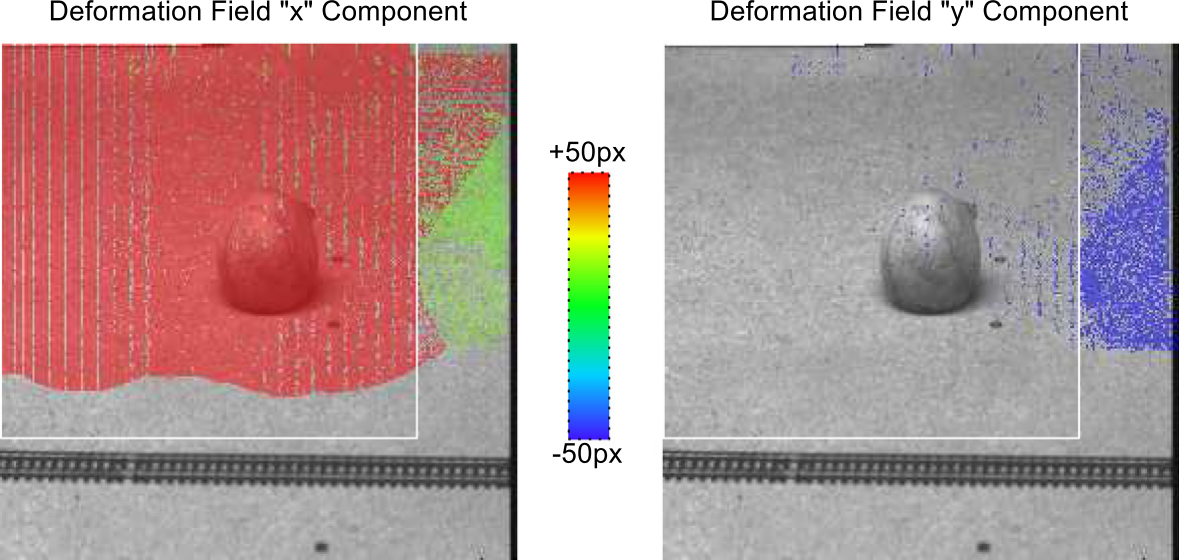
**Deformation field computed by NLS method.** Deformation field computed by NLS method (kindly provided by C. Xing) overlaid onto fixed image in “Ball” example.

We obtained the actual deformation field computed by the NLS algorithm and visualized it in Figure 2 by color-coding and overlaying the *x* and *y* components of the deformation vector at each pixel onto the fixed image. For convenience, the 156×196 pixel region that was shifted is also marked by a white box. The actual deformation field compares with the ground truth as follows: 

1. The boundary that separates the shifted from the stationary region at the bottom is curved and at a substantial distance from the true boundary.

2. In Region “C,” there is no ground truth, but the actual deformation is dominated by an area with a horizontal shift (which wraps around the image edge, via an undocumented periodic boundary condition), and a second large area with a dominant vertical shift. The boundary between them is curved and its location arbitrary.

3. Pixels with zero deformation are scattered throughout regions “A,” “B,” and “C” and even inside the principal object (ball).

These observations confirm our contention that the deformation field computed by the NLS method is largely arbitrary and cannot be relied upon for interpretation. A further significant observation is that the reformatted and difference images based on this deformation field and shown in Ref. [11] (Figures four(a) and five(a) therein) reveal no hint as to where the computed deformation is accurate with respect to the true deformation and where it is not.

### Image similarity fails as a measure of registration quality

To explain why the failure of NLS was not apparent from the results presented in Ref. [11] we performed “registrations” on the exact same image pairs used therein. To each image pair, we applied a rank-order permutation algorithm, “CURT” [12], the “Completely Useless Registration Tool.” In short, CURT sorts the pixels in each image in order of increasing intensity and maps each fixed image pixel to the moving image pixel at the equivalent index in the sort order. Thus, image similarity is maximized, but no meaningful spatial transformation is actually computed. Indeed, CURT was conceived specifically to demonstrate the inadequacy of image similarity for evaluating image registration.

The quantitative measures of image similarity achieved by CURT, as well as those achieved by NLS, are listed in Table 1: root residual mean squares (RRMS), cross correlation (CC), and entropy of image difference (EID). For all measures and all examples, CURT clearly outperformed NLS. Also, for all examples, the reformatted moving images are visually indistinguishable from the fixed images (see Additional file 1: Figure S1).

**Table 1:** Image similarities

		**NLS**^**∗**^				**CURT**		
**Example**		**RRMS**	**CC**	**EID**		**RRMS**	**CC**	**EID**
Ball		10.929	0.745	2.911		**0.000**	**0.962**	**0.000**
Bird		7.434	0.987	1.829		**0.2131**	**0.999**	**0.437**
Satellite		18.782	0.951	4.143		**9.416**	**0.999**	**2.562**
MRI		2.976	0.999	0.120		**0.002**	**1.000**	**0.017**

The best score (lowest for RRMS and EID, highest for CC) for each metric is printed in bold face. ^∗^NLS results were included as published in Ref. [11].

Thus, by the (flawed) reasoning employed in Ref. [11], CURT would have to be declared the far superior registration algorithm. Yet it is obvious from CURT’s design that it does not compute any valid spatial correspondence, i.e., it is not an effective registration algorithm. This demonstrates that image similarity cannot be used by itself to quantify image registration performance.

### Significance

The “Nonparametric Local Smoothing” algorithm [11] is not an effective registration technique. Correcting the research record on this matter is particularly important because the method was published in perhaps the most respected journal in computer science^a^. We made every effort to publish our results in that same journal but were unsuccessful (see Additional file 2).

Analysis of a deformation field computed by NLS revealed that it is largely arbitrary, not based in reality, and thus not suitable for the purposes of interpretation, quantification, and measurement. These issues are particularly salient in the case of high-dimensional registration where the number of parameters can match or even exceed the number of pixels in the image. In such cases, transformation regularity is fundamental to not only yielding a well-posed algorithm but also producing interpretable results. That is why the idea has persisted from early [13] to more current research in (especially dense, high-dimensional) image registration [14].

Although indeed continuous transformation models cannot represent discontinuous motion, we found that the NLS algorithm is unable to correctly recover such motion that actually *is* continuous. This is a significant shortcoming because motion involving actual, macroscopic physical objects must be at least locally continuous. In addition, while NLS is able to *represent* discontinuous motion in general, it is unable to determine the *correct* discontinuous motion (see Figures 1 and 2).

Our results demonstrate fundamental flaws in the NLS algorithm, resulting ultimately in its complete and utter failure to compute meaningful registrations. By using the exact data used by the algorithm’s authors, we have ensured that our evaluation satisfies the exact same assumptions made in the original paper. One particular such assumption is constancy of image intensities, as implied by the requirement *M*(*T*_1_(*x*,*y*),*T*_2_(*x*,*y*))=*R*(*x*,*y*) for the true transformation (*T*_1_,*T*_2_)(although one of the authors’ very own examples, “Satellite,” violates this assumption; see Additional file 3: Figure S2).

But intensity constancy is not even sufficient for this particular algorithm to work. In actual fact, the method requires that if $M(\hat{x},ŷ)=R(x,y)$, then $\hat{x}=T_{1}(x,y),ŷ=T_{2}(x,y)$, i.e., it requires that identity of image intensity (or here: of local patch texture) uniquely encodes the true correspondence between any fixed image pixel (or patch) and its location in the moving image. In other words, NLS implicitly assumes intensity (or patch) *uniqueness*, but this is simply not realistic in any conceivable application scenario. If it were realistic, then the “CURT” algorithm would compute valid registrations under these conditions as well, and as we have also demonstrated herein it would have to be considered superior to NLS.

### Conclusion

Xing & Qiu published an algorithm incorrectly assuming that maximizing image similarity is sufficient for image registration. The fact that accurate registration may improve image similarity metrics does not mean that optimizing similarity metrics necessarily leads to accurate registration which, with Xing and Qiu’s assistance, we have demonstrated experimentally herein.

It is tempting to blame the unconstrained and discontinuous transformation models of both NLS and CURT for the failure of image similarity to reflect the accuracy of spatial alignment. But even in the presence of an appropriate transformation model, “the desired optimum when registering images using voxel similarity measures is frequently not the global optimum, but is one of the local optima” [4]. This is the well-known issue of capture range – intensity-based registration typically converges to the “correct” solution only when initialized with a transformation within a certain neighborhood of the correct alignment. Thus, even with a perfectly appropriate and well-constrained transformation model, it is only within the capture range of the correct transformation that we can reasonably assume that improved image similarity corresponds to improved alignment.

In summary, it is imperative to understand that the goal of image registration is to establish spatial correspondence between images, often at a given specific scale, but *not* merely to make them look alike. Thus, as Crum *et al.*[15] noted, registration “validation tests the ability of registration to establish correspondence,” whereas image similarity is “uninformative about the magnitude of errors of correspondence.” The usefulness of the NLS algorithm for image registration was originally supported by experiments that ignored correspondence. But with correspondence now properly considered herein, we must conclude that NLS simply does not perform registration, which is a fatal flaw for a method specifically advertised as a registration algorithm.

## Endnote

^a^Ranked number one out of 108 journals in the “COMPUTER SCIENCE, ARTIFICIAL INTELLIGENCE” category of the 2010 Journal Citation Reports.

## Abbreviations

CC: Cross Correlation;CURT: Completely Useless Registration Tool;EID: Entropy of Image Difference;NLS: Nonparametric Local Smoothing

## Competing interests

The authors declare that they have no competing interests.

## Authors’ contributions

Both authors jointly conceived this study. TR obtained the image data used in the criticized paper, implemented the CURT algorithm, and ran it on the test data. BA implemented the ANTs registration software, and ran it on the test data. Both authors jointly wrote the text of this article and approved the final submitted manuscript.

## Supplementary Material

Additional file 1 **Supplemental Figure **1** – Results of “CURT” algorithm.** Results of “CURT” algorithm applied to images previously published in: C. Xing and P. Qiu, “Intensity-Based Image Registration by Nonparametric Local Smoothing,” IEEE Transactions on Pattern Analysis and Machine Intelligence, vol.33, no.10, pp. 2081–2092, Oct. 2011, doi: http://10.1109/TPAMI.2011.26. Ⓒ2011 IEEE. Reprinted, with permission, from IEEE Transactions on Pattern Analysis and Machine Intelligence. The “moving” and “fixed” images were kindly provided by C. Xing. Click here for file

Additional file 2 **Correspondence with IEEE-TPAMI.** This PDF document contains, in this order,
1. our Comment originally submitted to IEEE-TPAMI,
2. the notice of immediate rejection,
3. our request for reconsideration with detailed list of procedural and technical flaws in the editorial decision,
4. the final rejection notice, and
5. our comments on the final rejection notice (these were not submitted to TPAMI but are included here for clarification). Click here for file

Additional file 3 **Supplemental Figure **2** – “Satellite” Example.** Image intensity difference, not registration, carries information in Xing & Qiu’s “Satellite” example (Image source: http://webmodis.iis.u-tokyo.ac.jp/Landsat/). *Top row:* input satellite images. *Bottom row:* difference image and transparent red overlay of difference onto fixed image. Correcting changes in image intensity via spatial transformations (beyond affine alignment to match fields of view) has no basis in reality and violates the authors’ own stated assumption of intensity constancy.
Input satellite images were previously published in: C. Xing and P.Qiu, “Intensity-Based Image Registration by Nonparametric Local Smoothing,” IEEE Transactions on Pattern Analysis and Machine Intelligence, vol.33, no.10, pp. 2081–2092, Oct. 2011, doi: http://10.1109/TPAMI.2011.26. Ⓒ2011 IEEE. Reprinted, with permission, from IEEE Transactions on Pattern Analysis and Machine Intelligence. Click here for file

Additional file 4 Re-use permission for potentially IEEE-copyrighted materials. Click here for file
