# Supplementary material for: “Nonparametric Local Smoothing” is not image registration
Source: BMC Res Notes. 2012 Nov 1;5:610. doi: 10.1186/1756-0500-5-610 (PMC3740790; doi:10.1186/1756-0500-5-610)
Supplement: Additional file 1 — Supplemental Figure 1 – Results of “CURT” algorithm. Results of “CURT” algorithm applied to images previously published in: C. Xing and P. Qiu, “Intensity-Based Image Registration by Nonparametric Local Smoothing,” IEEE Transactions on Pattern Analysis and Machine Intelligence, vol.33, no.10, pp. 2081–2092, Oct. 2011, doi: http://10.1109/TPAMI.2011.26. Ⓒ2011 IEEE. Reprinted, with permission, from IEEE Transactions on Pattern Analysis and Machine Intelligence. The “moving” and “fixed” images were kindly provided by C. Xing. [file 1756-0500-5-610-S1.pdf]

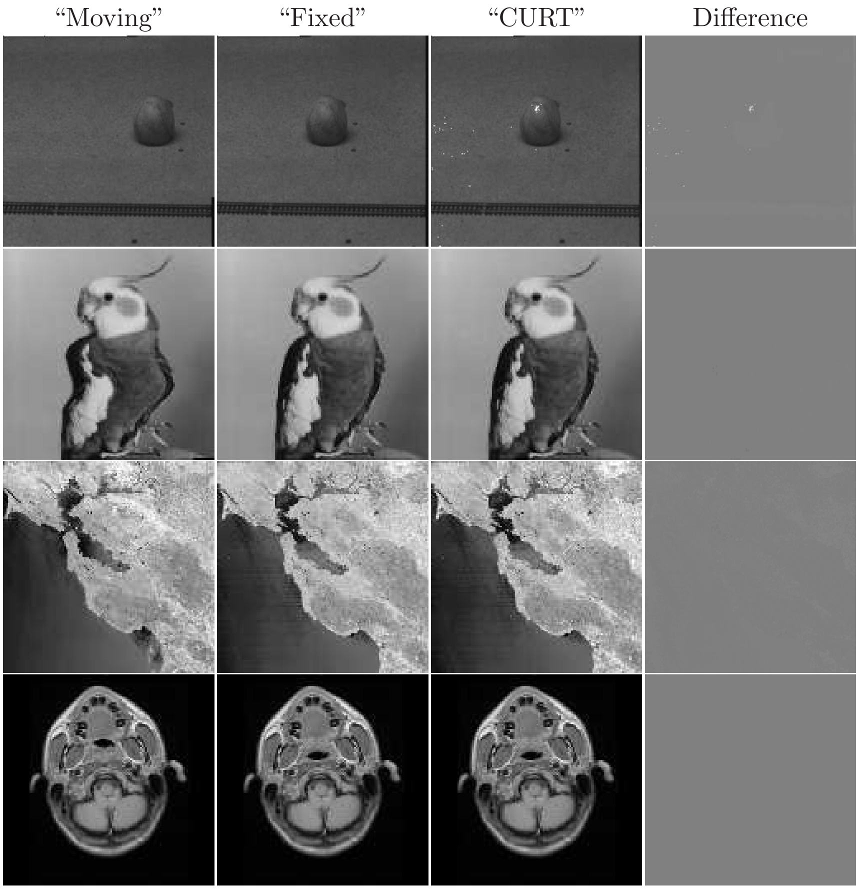

Supplemental Figure 1: Results of "CURT" algorithm applied to images previously published in: C. Xing and P. Qiu, "Intensity-Based Image Registration by Nonparametric Local Smoothing," IEEE Transactions on Pattern Analysis and Machine Intelligence, vol.33, no.10, pp. 2081–2092, Oct. 2011, doi: 10.1109/TPAMI.2011.26. ©2011 IEEE. Reprinted, with permission, from IEEE Transactions on Pattern Analysis and Machine Intelligence.
The "moving" and "fixed" images were kindly provided by C. Xing.
