# Supplementary material for: “Nonparametric Local Smoothing” is not image registration
Source: BMC Res Notes. 2012 Nov 1;5:610. doi: 10.1186/1756-0500-5-610 (PMC3740790; doi:10.1186/1756-0500-5-610)
Supplement: Additional file 2 — Correspondence with IEEE-TPAMI. This PDF document contains, in this order, 1. our Comment originally submitted to IEEE-TPAMI, 2. the notice of immediate rejection, 3. our request for reconsideration with detailed list of procedural and technical flaws in the editorial decision, 4. the final rejection notice, and 5. our comments on the final rejection notice (these were not submitted to TPAMI but are included here for clarification). [file 1756-0500-5-610-S2.pdf]

Comment submitted to IEEE Transactions on Pattern Analysis and Machine Intelligence (IEEE-TPAMI)

March 5, 2012.

Comment on "Intensity-based image registration by nonparametric local smoothing"

Torsten Rohlfing, *Member, IEEE* and Brian Avants

***Abstract*—A recent article by Xing & Qiu (IEEE-TPAMI 33(10):2081–2092, 2011) is based on an inappropriately narrow conceptualization of the image registration problem as the task of making two images look alike, which disregards whether the established spatial correspondence is plausible. Xing & Qiu use image similarities alone as a measure of registration performance, but these measures do not relate reliably to the realism of the correspondence map. We show experimentally that the method proposed by Xing & Qiu is not an effective registration algorithm, despite optimizing image similarity, as it does not compute accurate, interpretable transformations. Even judged by image similarity alone, the method is consistently outperformed by a simple pixel permutation algorithm. Our results emphasize the need to apply registration evaluation criteria that are sensitive to whether correspondences are accurate and mappings between images are physically interpretable. These goals cannot be achieved by simply reporting image similarities.**

***Index Terms*—image registration; correspondence; accuracy**

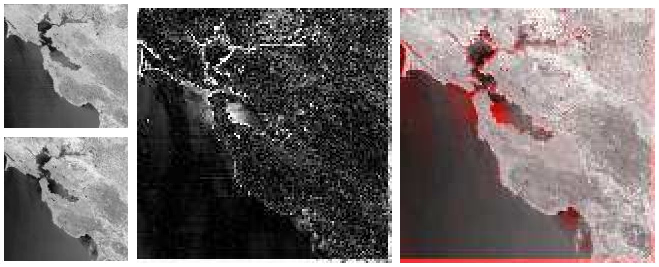

Fig. 1. Image intensity difference, not registration, carries information in the "Satellite" example from [7]. *Left:* input images. *Center:* difference image. *Right:* transparent red overlay of difference onto fixed image.

I. Introduction

IMAGE REGISTRATION is "a process for determining the correspondence of features between images" [1], "the determination of a one-to-one mapping or transformation between the coordinates in one space and those in another" [2], and with the objective to "bring the modalities involved into spatial alignment" [3]. Registration "geometrically aligns two images" [4], thus "determining the spatial alignment between images" [5].

What is *not* the goal of registration, however, is to make one ("moving") image appear maximally like another ("fixed") image. If this were the case, nothing would be gained by the process of registration, as we are given *a priori* the fixed image. Instead, the primary result of registration is the correspondence established between the images. A useful geometric transformation between images must allow *interpretation* of their differences and, potentially, statistics in a well-defined transformation space [6]. Performance measures of image registration, therefore, must consider the specific properties of correspondences in addition to the numerical similarity achieved between the images.

In their recent paper, "Intensity-based image registration by nonparametric local smoothing," Xing & Qiu [7] are thus incorrect to state, "the major goal of image registration is to find a geometrical transformation $\mathbf{T}(x,y) = (T_1(x,y), T_2(x,y))$ such that $Z_M(\mathbf{T}(x,y))$ is as close to $Z_R(x,y)$ as possible." This optimization problem represents a common computational tool to compute the registration, but it is not its goal. Instead, the fundamental goal of image registration is to find a transformation $\mathbf{T}$ such that the difference $||\mathbf{T}-\mathbf{T}_{\text{true}}||$ between estimated and true transformation is minimized. This, however, cannot be determined based on image similarity.

Consider the "Satellite" example from Ref. [7] in Fig. 1. Differences between the images represent changes in image contrast, not movement in space. Areas of water have changed optical properties but are still in exactly the same location. To explain such stationary intensity differences with a spatial transformation computed by a registration algorithm is simply misguided. Yet this misconception of the purpose of image registration is the foundation of Xing & Qiu's [7] experiments that rely on image similarity alone to quantify registration quality. But image similarity is not a valid measure of registration accuracy [9], thus leaving unsupported the conclusion that "Nonparametric Local Smoothing" (NLS) performs effective image registration.

More importantly, in this Comment we provide *direct* and *specific* evidence that the experimental design and reasoning employed by Xing & Qiu [7] are flawed and that their NLS algorithm is not suitable for image registration at all by experimentally substantiating two crucial observations. Firstly, a simple permutation-based algorithm outperforms the NLS method by all criteria used in by Xing & Qiu. Second, a deformation field computed by the NLS method is largely arbitrary and substantially different from the ground truth.

T. Rohlfing is with the Neuroscience Program at SRI International, 333 Ravenswood Avenue, Menlo Park, CA 94025-3493, USA. Phone: +1-650-859-3379, fax: +1-650-859-2743 (e-mail: rohlfing@ieee.org).

B. Avants is with Penn Image Computing and Science Laboratory (PICSL), Department of Radiology, University of Pennsylvania School of Medicine, Philadelphia, PA 19104 USA.

0000–0000/00$00.00 © 201X IEEE

TABLE I
IMAGE SIMILARITIES

Example	NLS*			CURT		
	RRMS	CC	EID	RRMS	CC	EID
Ball	10.929	0.745	2.911	**0.000**	**0.962**	**0.000**
Bird	7.434	0.987	1.829	**0.2131**	**0.999**	**0.437**
Satellite	18.782	0.951	4.143	**9.416**	**0.999**	**2.562**
MRI	2.976	0.999	0.120	**0.002**	**1.000**	**0.017**

*NLS results taken from Ref [7]. The best score (lowest for RRMS and EID, highest for CC) for each metric is printed in bold face.

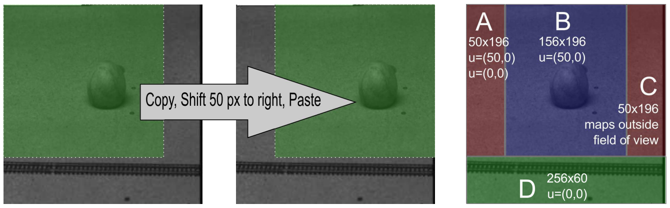

Fig. 2. Illustration of reconstructed procedure to generate moving image (*left*) and corresponding ground truth deformation field (*right*) for "Ball" example.

II. IMAGE SIMILARITY FAILS AS A MEASURE OF REGISTRATION QUALITY

We obtained the identical images used in Ref. [7] and shown in Figures 2, 6, 8, and 10 therein. For each image pair, we performed registrations using a rank-order permutation algorithm, "CURT" [9], the "Completely Useless Registration Tool." In short, CURT sorts the pixels in each image by increasing intensity and maps each fixed image pixel to the moving image pixel at the equivalent index in the sort order. Thus, image similarity is maximized, but no meaningful spatial transformation is actually computed. Indeed, CURT was conceived specifically to demonstrate the inadequacy of image similarity for evaluating image registration.

The quantitative measures of image similarity are listed in Table I: root residual mean squares (RRMS), cross correlation (CC), and entropy of image difference (EID). For all measures and all examples, CURT clearly outperforms NLS. Also, for all examples, the warped images are visually indistinguishable from the fixed images (see Supplemental Data).

Thus, by the (flawed) reasoning employed in Ref. [7], CURT would have to be declared the far superior registration algorithm. Yet it is obvious from CURT's design that it does not compute any valid spatial correspondence, i.e., it is not an effective registration algorithm. This demonstrates that image similarity cannot be used to quantify image registration performance.

III. THE NLS METHOD DOES NOT CREATE INTERPRETABLE TRANSFORMATIONS

From the images of the "Ball" example, we reconstructed the ground truth transformation between them as follows. We determined that the top-left area, 156×196 pixels, of the fixed image had been copied, shifted by 50 pixels, and pasted into the top-right image corner (Fig. 2). The ground truth deformation field is thus partitioned into four distinct regions: "A," duplicated content, two equally correct mappings, $u = (0, 0)$ and $u = (50, 0)$; "B," $u = (50, 0)$. "C," content pasted over, transformation undefined; "D," unmoved, $u = (0, 0)$.

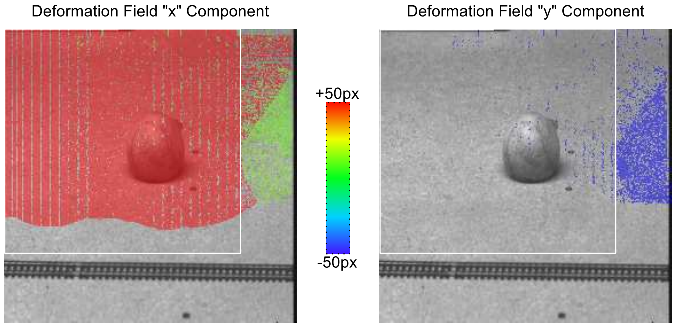

Fig. 3. Deformation field computed by NLS method (kindly provided by C. Xing) overlaid onto fixed image in "Ball" example.

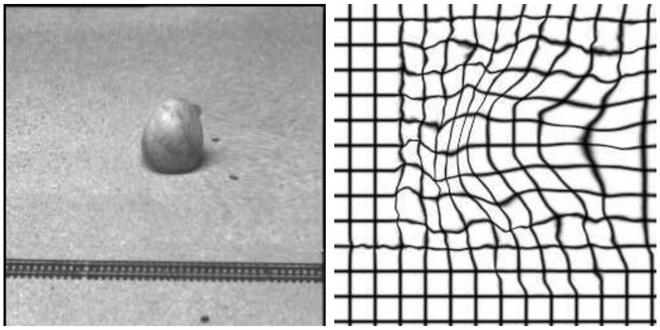

Fig. 4. Reformatted image and deformation field computed using diffeomorphic registration algorithm [10].

We obtained the actual deformation field computed by the NLS algorithm and visualized it in Fig. 3 by color-coding and overlaying the x and y components of the deformation vector at each pixel onto the fixed image. For convenience, the 156×196 pixel region that was shifted is also marked by a white box. The actual deformation field compares with the ground truth as follows:

1) The boundary that separates the shifted from the stationary region at the bottom is curved and at a substantial distance from the true boundary.
2) In Region "C," there is no ground truth, but the actual deformation is dominated by an area with a horizontal shift (which wraps around the image edge, via an undocumented periodic boundary condition), and a second large area with a dominant vertical shift. The boundary between them is curved and its location arbitrary.
3) Pixels with zero deformation are scattered throughout regions "A," "B," and "C" and even inside the principal object (ball).

These observations confirm our contention that the deformation field computed by the NLS method is largely arbitrary and cannot be relied upon for interpretation.

By contrast, consider the reformatted image and deformation field computed using a diffeomorphic registration algorithm [10]. Although the resulting reformatted image is not so similar to the fixed image as results from the NLS or CURT algorithms, the deformation field is free of discontinuities and yet appears to model the discontinuous separation between the "Ball" and "Railway" areas at least as faithfully as does NLS (compare Fig. 3).

IV. Conclusion

The “Nonparametric Local Smoothing” algorithm [7] is not an effective registration technique. By all image-based criteria used in Ref. [7], NLS was outperformed by a simple permutation algorithm [9], which is known to not compute valid spatial correspondences. Further, analysis of a deformation field computed by NLS revealed that it is largely arbitrary, not based in reality, and thus not suitable for the purposes of interpretation, quantification, and measurement.

These issues are particularly salient in the case of high-dimensional registration where the number of parameters can match or even exceed the number of pixels in the image. In such cases, transformation regularity is fundamental to not only yielding a well-posed algorithm but also producing interpretable results. That is why the idea has persisted from early [11] to more current research in (especially dense, high-dimensional) image registration [12].

Although indeed continuous transformation models cannot represent discontinuous motion, we found that the NLS algorithm is unable to correctly recover such motion that actually *is* continuous. This is a significant shortcoming because motion involving actual, macroscopic physical objects must be at least locally continuous. In addition, while NLS is able to *represent* discontinuous motion in general, it is unable to determine the *correct* discontinuous motion (see Figs. 2 and 3).

It cannot be emphasized enough that the goal of image registration is to establish spatial correspondence between images, often at a given specific scale, but *not* merely to make them look alike. Thus, as Crum *et al.* [13] noted, registration “validation tests the ability of registration to establish correspondence,” whereas image similarity is “uninformative about the magnitude of errors of correspondence.”

Acknowledgments

C. Xing kindly provided the images used in Ref. [7] as well as the deformation field generated by the NLS method for the “Ball” example.

TR was supported by the NIH-NIBIB, Grant No. EB008381.

References

[1] W.R. Crum, T. Hartkens, and D.L.G. Hill, “Non-rigid image registration: theory and practice,” *Br. J. Radiol.*, vol. 77, pp. S140–153, 2004.

[2] C.R. Maurer, Jr., J.M. Fitzpatrick, M.Y. Wang, R.L. Galloway, Jr., R.J. Maciunas, and G.S. Allen, “Registration of head volume images using implantable fiducial markers,” *IEEE Trans. Med. Imag.*, vol. 16, no. 4, pp. 447–462, Aug. 1997.

[3] J.B.A. Maintz and M.A. Viergever, “A survey of medical image registration,” *Med. Image. Anal.*, vol. 2, no. 1, pp. 1–36, 1998.

[4] B. Zitová and J. Flusser, “Image registration methods: a survey,” *Image Vision Comput.*, vol. 21, no. 11, pp. 977–1000, Oct. 2003.

[5] D.L.G. Hill, P.G. Batchelor, M. Holden, and D.J. Hawkes, “Medical image registration,” *Phys. Med. Biol.*, vol. 46, pp. R1–R45, Mar. 2001.

[6] J. Ashburner, C. Hutton, R. Frackowiak, I. Johnsrude, C. Price, and K. Friston, “Identifying global anatomical differences: Deformation-based morphometry,” *Hum. Brain Map.*, vol. 6, no. 5–6, pp. 348–357, 1998.

[7] C. Xing and P. Qiu, “Intensity-based image registration by nonparametric local smoothing,” *IEEE Trans. Pattern Anal. Machine Intell.*, vol. 33, no. 10, pp. 2081–2092, Oct. 2011.

[8] T. Rohlfing, C.R. Maurer, Jr., D.A. Bluemke, and M.A. Jacobs, “Volume-preserving nonrigid registration of MR breast images using free-form deformation with an incompressibility constraint,” *IEEE Trans. Med. Imag.*, vol. 22, no. 6, pp. 730–741, Jun. 2003.

[9] T. Rohlfing, “Image similarity and tissue overlaps as surrogates for image registration accuracy: Widely used but unreliable,” *IEEE Trans. Med. Imag.*, vol. 31, no. 2, pp. 153–163, Feb. 2012.

[10] B. Avants, C. Epstein, M. Grossman, and J. Gee, “Symmetric diffeomorphic image registration with cross-correlation: Evaluating automated labeling of elderly and neurodegenerative brain,” *Med. Image. Anal.*, vol. 12, no. 1, pp. 26–41, Feb. 2008.

[11] B.K.P. Horn and B.G. Schunck, “Determining optical flow,” *Artif. Intell.*, vol. 17, pp. 185–203, 1981.

[12] B.T. Yeo, M.R. Sabuncu, R. Desikan, B. Fischl, and P. Golland, “Effects of registration regularization and atlas sharpness on segmentation accuracy,” *Med. Image. Anal.*, vol. 12, no. 5, pp. 603–615, Oct. 2008.

[13] W.R. Crum, L.D. Griffin, D.L.G. Hill, and D.J. Hawkes, “Zen and the art of medical image registration: correspondence, homology, and quality,” *NeuroImage*, vol. 20, no. 3, pp. 1425–1437, Nov. 2003.

Notice of Immediate Rejection

received

March 10, 2012.

RE: TPAMI-2012-03-0163, "Comment on "Intensity-based image registration by nonparametric local smoothing""
Manuscript Type: Comments

Dear Dr. Torsten Rohlfing,

Thank you for your submission to the IEEE Transactions on Pattern Analysis and Machine Intelligence.

I have reviewed your manuscript, and determined that it has significant deficiencies which prohibit us from processing it any further.

For guidance on the appropriate content for submission to the IEEE Transactions on Pattern Analysis and Machine Intelligence, please refer to the inside back cover of Transactions on Pattern Analysis and Machine Intelligence under "Scope of the Journal," or on the web at:

http://computer.org/TPAMI/author_new.htm

We are aware of the effort that has gone into your paper, and hope that you will consider us for future submissions more compatible with TPAMI objectives.

I thank you for your interest in the IEEE Transactions on Pattern Analysis and Machine Intelligence.

Thank you,

Dr. Sing Bing Kang,
AEIC
IEEE Transactions on Pattern Analysis and Machine Intelligence

=========================

Dear Authors,

Comment papers are rare in IEEE TPAMI, and only very significant insights on published papers are accepted. The original paper uses the brightness constancy assumption; its novelty is more in the use of nonparametric geometric transformation rather than being able to register images with very different appearances. So, while the comments are valid, they are not significant. It is well known that there are alternative techniques for registering images that look very different (in addition to what was cited), e.g., using mutual information (see, for example, Pluim et al. (2003)) and invariant features (see, for example, Yang et al. (2007)).

Additional references:

* J.P.W. Pluim, J.B.A. Maintz, M.A. Viergever, "Mutual Information Based Registration of Medical Images: A Survey," IEEE Transactions on Medical Imaging, 22(8):986-1004 , July 2003.
* G. Yang, C.V. Stewart, M.Sofka, and C.-L. Tsai, "Registration of Challenging Image Pairs: Initialization, Estimation, and Decision," IEEE TPAMI, 29(11):1973-1989, Nov. 2007.

=========================

Request to Reconsider

sent to IEEE-TPAMI

March 14, 2012.

Dear Dr. Zabih:

We request reconsideration and reversal of the decision on our comment TPAMI-2012-03-0163, primarily on the grounds that *proper editorial policy was not followed.*

As stated at http://www.computer.org/portal/web/peerreviewjournals/editor#comments,

> "The editor reviews the comments paper and if they believe the commenting authors *may have a valid point*, they contact the previously published paper's authors with an invitation to review and respond with a rebuttal."

(Emphasis added).

The AEIC admits specifically that our comments are indeed valid. Therefore, the Comment must be sent to the previous paper's authors. This has apparently not happened, so the decision to dismiss our Comment is premature.

In addition, we would like to point out the following issues to support our request for reconsideration and to ensure a fair and well-informed decision procedure:

<u>1. Our Comment if fully within Scope</u>

The aforementioned policy defines a Comment as "a paper commenting on an error one has found or a disagreement one has with a previously published paper." Our Comment demonstrates that *the previously published method does not do what it claims to do*, namely perform effective image registration. This constitutes an error in the previous paper (and we certainly have a disagreement with it), and thus our Comment is fully within the scope of TPAMI Comment papers.

Furthermore, it should be in TPAMI's own interest to ensure the accuracy of the scientific record by correcting previously published materials when necessary.

<u>2. Our Comment is Significant</u>

We are clearly demonstrating, and this is undisputed so far, that a paper previously published in TPAMI is *technically substantially flawed*. Given TPAMI's excellent reputation, we assume that this is not a common situation. Therefore, our demonstration of substantial flaws is a significant insight. (If this wasn't significant then what insight possibly could be?)

<u>3. We Make Identical Assumptions as the Original Article</u>

The AEIC's evaluation suggests that we failed to consider the assumptions that the previous paper is based on. But we are *using the exact data used by the authors* - thus, our demonstration satisfies the same assumptions, and the proposed method is still broken.

Also, when we carefully consider the assumptions, intensity constancy is not really what the criticised method is truly based upon. Instead, it is based on "intensity uniqueness", i.e., in order for it to be effective, image intensity must uniquely encode corresponding features. This is assumption, however, is impossible to satisfy, which is why the algorithm does not compute valid registrations.

4. Novelty of a Flawed Method is Irrelevant

The AEIC's evaluation suggests that the area we are criticizing is not the primary contribution of the previous paper. But we are criticizing that the method is ineffective - it simply does not achieve its stated goal. What part of the method, if any, is innovative is irrelevant if the method is fundamentally broken.

5. Alternative Methods are Irrelevant

Indeed, as stated by the AEIC, there are various registration methods in the literature that are designed to address different application scenarios. But as our Comment shows, the criticized method is *not effective in any application*, including the ones used in the paper itself. To cite other methods for other applications is, therefore, no more than a red herring and distraction from the actual issue, which is the flawed nature of the particular algorithm published in TPAMI.

6. The AEIC is not an Expert

With all due respect, according to his own online publication list Dr. Kang has very few (four, to be precise) publications in the field of registration, and none at all since 1999. At the same time, TPAMI has at least three AEs who are outstanding experts in this field (Drs. Golland, Stewart, and Cootes), and we request that one of them be asked instead to evaluate our Comment.

7. Potential Conflicts of Interest

It seems that the standard procedure for handling Comments submitted to TPAMI involves judgements by a) the handling AE of the original paper, b) its authors, and c) the original reviewers. To be in favour of publishing the Comment, each and every one of these would have to admit to having made a mistake, either of technical nature or of judgement.

In other words, *not a single person involved in the standard decision process is without a potential conflict of interest*. While we obviously cannot, and do not, assert that this is an actual problem for this particular Comment, we observe that it is generally accepted practice to avoid even the appearance of a conflict of interest. We therefore request that at the very least a different AE be asked to handle our Comment than handled the original paper, and that at least one additional, independent and qualified reviewer also be invited to comment.

We appreciate your attention and consideration.

Sincerely,
Dr. Torsten Rohlfing

Notice of Upheld Rejection

received

March 17, 2012.

Dear Dr. Rohlfing,

I have considered the issues that you raise and decided to uphold the original decision to reject this paper.

As an initial matter, rejection early in the process (which, like all editorial decisions, is purely at the discretion of the EIC) is in fact intended to help authors by rapidly giving them an opportunity to submit to a more appropriate venue. TPAMI gets nearly 1,000 submissions per year and rejects the vast majority due to our limited page budget. As a consequence the EIC and AEIC's have considerable experience as to what papers will be likely to be accepted or not. While it is always painful to have a paper rejected, it is very much in your interests to obtain a quick decision, since in our considered judgment if we sent this out for review the same outcome would occur after months of delay.

In terms of procedure, I realize that the way in which TPAMI issues decisions is a bit confusing. It is not unreasonable for you to assume that the decision was made by the AEIC who signed it, but in fact AEIC Sing Bing Kang discussed this internally with myself and with other senior researchers.

As to the content of your submission, I have personally chosen to limit the number of comments papers to only include those that are likely to have wide appeal to the TPAMI readership. In this particular case, the consensus among myself and the senior people who discussed your submission was that it did not meet this (very high) bar. In our view the comment paper is addressing a feature that the original paper never claimed or tried to demonstrate. The technique in the original paper assumes brightness constancy (the title does include the phrase “Intensity Based Image Registration”), and naturally will not work on any image pair with substantial changes in intensity. The comment is not correcting claims in the other paper, but rather pointing out an obvious limitation.

There are many alternative venues where you could publish your critique. While unfortunately we have not been able to accommodate this in TPAMI, I am sure that you will find a suitable journal.

Sincerely,
Ramin Zabih
Editor-in-Chief, TPAMI

Comments on EiC Decision after Reconsideration

We note that the EiC does not address any of the issues pointed out in our Request for Reconsideration, but merely repeats flawed reasoning.

1. Our analysis is based on the exact same data used by the original authors, so demonstration that the algorithm does not work on these data does very much disprove a claim made in the original article.

2. “Intensity-based registration” does not at all mean “registration in the presence of constant intensities,” but rather, is the opposite of “feature-based registration.” This demonstrates again that the decision to reject our Comment was made by non-experts who simply do not understand image registration (or even know its terminology).
