# Supplementary material for: “Nonparametric Local Smoothing” is not image registration
Source: BMC Res Notes. 2012 Nov 1;5:610. doi: 10.1186/1756-0500-5-610 (PMC3740790; doi:10.1186/1756-0500-5-610)
Supplement: Additional file 3 — Supplemental Figure 2 – “Satellite” Example. Image intensity difference, not registration, carries information in Xing & Qiu’s “Satellite” example (Image source: http://webmodis.iis.u-tokyo.ac.jp/Landsat/). Top row: input satellite images. Bottom row: difference image and transparent red overlay of difference onto fixed image. Correcting changes in image intensity via spatial transformations (beyond affine alignment to match fields of view) has no basis in reality and violates the authors’ own stated assumption of intensity constancy. Input satellite images were previously published in: C. Xing and P.Qiu, “Intensity-Based Image Registration by Nonparametric Local Smoothing,” IEEE Transactions on Pattern Analysis and Machine Intelligence, vol.33, no.10, pp. 2081–2092, Oct. 2011, doi: http://10.1109/TPAMI.2011.26. Ⓒ2011 IEEE. Reprinted, with permission, from IEEE Transactions on Pattern Analysis and Machine Intelligence. [file 1756-0500-5-610-S3.pdf]

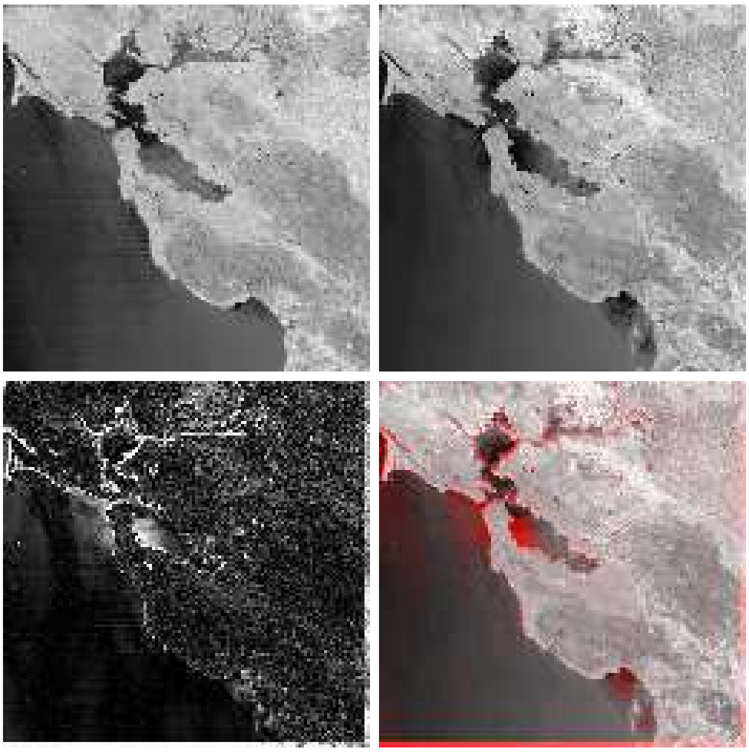

Supplemental Figure 2: Image intensity difference, not registration, carries information in Xing & Qiu's "Satellite" example (Image source: `http://webmodis.iis.u-tokyo.ac.jp/Landsat/`). *Top row:* input satellite images. *Bottom row:* difference image and transparent red overlay of difference onto reference image. Correcting changes in image intensity via spatial transformations (beyond affine alignment to match fields of view) has no basis in reality and violates the authors' own stated assumption of intensity constancy..
Input satellite images were previously published in: C. Xing and P. Qiu, "Intensity-Based Image Registration by Nonparametric Local Smoothing," IEEE Transactions on Pattern Analysis and Machine Intelligence, vol.33, no.10, pp. 2081–2092, Oct. 2011, doi: 10.1109/TPAMI.2011.26. ©2011 IEEE. Reprinted, with permission, from IEEE Transactions on Pattern Analysis and Machine Intelligence.
