# Supplementary material for: “Nonparametric Local Smoothing” is not image registration
Source: BMC Res Notes. 2012 Nov 1;5:610. doi: 10.1186/1756-0500-5-610 (PMC3740790; doi:10.1186/1756-0500-5-610)
Supplement: Additional file 4 — Re-use permission for potentially IEEE-copyrighted materials. [file 1756-0500-5-610-S4.pdf]

IEEE LICENSE
TERMS AND CONDITIONS

Jun 15, 2012

This is a License Agreement between Torsten Rohlfing ("You") and IEEE ("IEEE") provided by Copyright Clearance Center ("CCC"). The license consists of your order details, the terms and conditions provided by IEEE, and the payment terms and conditions.

License Number	2930310016913
License date	Jun 15, 2012
Licensed content publisher	IEEE
Licensed content publication	Pattern Analysis and Machine Intelligence, IEEE Transactions on
Licensed content title	Intensity-Based Image Registration by Nonparametric Local Smoothing
Licensed content author	Chen Xing; Peihua Qiu
Licensed content date	Oct. 2011
Volume number	33
Issue number	10
Type of Use	Journal/Magazine
Requestor type	non-commercial/non-profit
IEEE Member	yes
IEEE Member ID	80181554
Author of this new paper	yes
Format	electronic
Portion	figures/tables/graphs
Number of figures/tables /graphs	3
Figures/tables/graphs to be used	Raw images in Fig. 2 without annotations (not the figure itself). Raw images in Fig. 6. Raw images in Fig. 10. Also raw images in Fig. 8 without annotation, but these are from a publicly available, third-party source (University of Tokyo)
Will you be translating?	no
Circulation/distribution	99999999
Order reference number	
Title of the article	"Nonparametric local smoothing" is not image registration
Publication the new article is in	BMC Research Notes
Publisher of the article	BioMed Central
Author of new article	Torsten Rohlfing & Brian Avants
Expected publication date	Sep 2012
Estimated size of the article (pages)	4
Billing Type	Credit Card

Credit card info	American Express ending in 3007
Credit card expiration	08/2014
Total	3.50 USD
Terms and Conditions	

TERMS AND CONDITIONS FOR REUSE OF IEEE MATERIAL SELECTED FOR LICENSING (THE "LICENSED MATERIAL") BASED ON “TYPE OF USE” AND “FORMAT” SELECTED FOR LICENSING BY USER

By clicking "accept" in connection with completing this licensing transaction, you, as "User" do agree that the following terms and conditions apply to the use of the material you selected for licensing (the "Licensed Material"), along with the Billing and Payment terms and conditions established by Copyright Clearance Center, Inc. ("CCC"), at the time that you opened your RightsLink account and that are available at any time at http://myaccount.copyright.com .

Grant of Limited License

IEEE hereby grants to you a non-exclusive, non-transferable worldwide license to use the Licensed Material in the "TYPE OF USE" and "FORMAT" that you outlined in the RightsLink form in connection with this transaction, and as outlined in accordance with the terms and conditions of this Agreement. This license does not include any photography, illustrations or advertisements that may appear in connection with the Licensed Material and does not extend to any revision or subsequent edition in which the Licensed Material may appear.

Types of Use

Complete terms and conditions and "TYPES OF USE" can be found in the License Agreement that will be available to you during the online order process. Certain terms of use in some IEEE licenses may take precedence over and supersede certain rights granted through RightsLink services. IEEE also reserves the right to restrict the types and total number of items that may be reused in any type of publication or medium. For additional information on the types of use available by IEEE and through RightsLink, please refer to http://www.ieee.org/publications_standards/publications/rights/rightslink_usetypes.html .

Authorized Use

The license granted is granted for a one-time use for REPUBLICATION IN THE "TYPE OF USE" and "FORMAT" that you outlined in the RightsLink form in connection with this transaction, to be completed within one (1) year from the date upon which this license is effective, with a maximum distribution equal to the number that you identified in the RightsLink form in connection with this transaction.

Restrictions on Use

All uses not specifically authorized in this license and specified in the options for reusing IEEE Licensed Material available through the RightsLink service are prohibited, including (i) altering or modifying the Licensed Material in any manner, translating the Licensed Material into another language or creating any derivative work based on the Licensed Material; (ii) storing or archiving the Licensed Material in any electronic medium or in any form now invented or

devised in the future, except where permission is granted to do so. For additional information on the types of use available by IEEE and through RightsLink, please refer to http://www.ieee.org/publications_standards/publications/rights/rightslink_usetypes.html .

If the Licensed Material is altered or modified in any manner, it must be within the scope of the license granted and it must not alter the meaning of the Licensed Material or in any way reflect negatively on the IEEE or any writer of the Licensed Material.

Any use of the Licensed Material in any way that would be considered libelous, defamatory, abusive or obscene, in violation of any applicable law or the proprietary rights of a third party and or used in connection with the advertising or promotion of any product or service is also prohibited.

You agree to use your best efforts to prevent unauthorized use of the Licensed Material.

License Effective Only Upon Payment and Author Approval

The license granted to you is effective only upon (i) receipt of full payment from you as provided in CCC's Billing and Payment terms and conditions and (ii) your having obtained the author's approval of your proposed use of the Licensed Material as described here.

IEEE Intellectual Property Rights

You agree that IEEE is the owner of all right, title and interest in the Licensed Material and/or has the right to license the Licensed Material, including all copyright rights and other intellectual property rights under United States and international law.

Termination

In the event that you breach any of these terms and conditions or any of CCC's Billing and Payment terms and conditions, the license granted herein shall be terminated immediately. Any use of the Licensed Material after termination, as well as any use of the Licensed Material beyond the scope of these terms and conditions, may constitute copyright infringement and IEEE reserves the right to take any and all action to protect its rights in the Licensed Material.

Copyright Notice

You must include the following copyright and permission notice (WITH DETAILS FILLED IN BY YOU) in connection with the Authorized Use of the Licensed Material:

© [Year] IEEE. Reprinted, with permission, from [complete publication information].

Warranty and Indemnity

You warrant that you have all rights necessary to enter into this agreement and hereby indemnify and agree to hold harmless IEEE and CCC, and their respective officers, directors, employees and agents, from and against any and all claims arising out of your use of the Licensed Material other than as specifically authorized pursuant to this license.

Limited Warranty and Limitation of Liability

THE RIGHT TO USE THE LICENSED MATERIAL IS GRANTED ON AN "AS IS" BASIS AND IEEE MAKES NO WARRANTY, EXPRESS OR IMPLIED WITH RESPECT TO THE LICENSED MATERIAL, INCLUDING ALL WARRANTIES OF QUALITY, ACCURACY AND/OR FITNESS FOR A PARTICULAR PURPOSE, AND IEEE SHALL NOT BE LIABLE UNDER ANY CIRCUMSTANCES FOR ANY LOSSES RESULTING FROM YOUR RELIANCE ON OR USE OF ANY INFORMATION CONTAINED IN THE LICENSED MATERIAL.

No Transfer or Assignment of License

This license is personal to you and may not be sublicensed, assigned, or transferred by you to any other person without IEEE's written permission.

Objection to Contrary Terms

IEEE hereby objects to any terms contained in any purchase order, acknowledgment, check endorsement or other writing prepared by you, which terms are inconsistent with these terms and conditions or CCC's Billing and Payment terms and conditions. These terms and conditions, together with CCC's Billing and Payment terms and conditions (which are incorporated herein), comprise the entire agreement between you and IEEE concerning this licensing transaction. In the event of any conflict between your obligations established by these terms and conditions and those established by CCC's Billing and Payment terms and conditions, these terms and conditions shall control.

Payment Terms

If you would like to pay for this license now, please remit this license along with your payment made payable to "COPYRIGHT CLEARANCE CENTER" otherwise you will be invoiced within 48 hours of the license date. Payment should be in the form of a check or money order referencing your account number and this invoice number. Payments should be sent to the address noted below:

Copyright Clearance Center
Dept 001
P.O. Box 843006
Boston, MA 02284-3006

Once you receive your invoice for this order, you may pay by credit card. Additional information is provided to users at the time their credit card order is placed.

For suggestions or comments regarding this order, contact RightsLink Customer Support: customercare@copyright.com or +1-877-622-5543 (toll free in the US) or +1-978-646-2777.

Comments and Questions for IEEE

All comments and/or questions related to RightsLink permission services or comments and questions regarding IEEE licensing policies should be sent to discoverservices@ieee.org. Users may also call Author Support & Content Discovery staff at 732.562.3965.

A copy of both the license and these terms should be retained for your files.

Other Terms and Conditions: None

Updated 12/2011 nbd-IEEE

If you would like to pay for this license now, please remit this license along with your payment made payable to "COPYRIGHT CLEARANCE CENTER" otherwise you will be invoiced within 48 hours of the license date. Payment should be in the form of a check or money order referencing your account number and this invoice number RLNK500800306.
Once you receive your invoice for this order, you may pay your invoice by credit card. Please follow instructions provided at that time.

Make Payment To:
Copyright Clearance Center
Dept 001
P.O. Box 843006
Boston, MA 02284-3006

For suggestions or comments regarding this order, contact RightsLink Customer Support: customercare@copyright.com or +1-877-622-5543 (toll free in the US) or +1-978-646-2777.

Gratis licenses (referencing $0 in the Total field) are free. Please retain this printable license for your reference. No payment is required.
